# Circulating tumor cells detected by lab-on-a-disc: Role in early diagnosis of gastric cancer

**DOI:** 10.1371/journal.pone.0180251

**Published:** 2017-06-29

**Authors:** Hwa Mi Kang, Gwang Ha Kim, Hye Kyung Jeon, Dae Hwan Kim, Tae Yong Jeon, Do Youn Park, Hyunjin Jeong, Won Joo Chun, Mi-Hyun Kim, Juhee Park, Minji Lim, Tae-Hyeong Kim, Yoon-Kyung Cho

**Affiliations:** 1 Department of Internal Medicine, Pusan National University School of Medicine and Biomedical Research Institute, Pusan National University Hospital, Busan, Korea; 2 Department of Surgery, Pusan National University School of Medicine, Busan, Korea; 3 Department of Pathology, Pusan National University School of Medicine, Busan, Korea; 4 Center for Soft and Living Matter, Institute for Basic Science (IBS), Ulsan, Korea; 5 Department of Biomedical Engineering, School of Life Sciences, Ulsan National Institute of Science and Technology (UNIST), Ulsan, Korea; Hunter College, UNITED STATES

## Abstract

**Background:**

The use of circulating tumor cells (CTCs) as an early diagnostic biomarker and prognostic indicator after surgery or chemotherapy has been suggested for various cancers. This study aimed to evaluate CTCs in patients who underwent gastrectomy for gastric cancer and to explore their clinical usefulness in the early diagnosis of gastric cancer.

**Methods:**

A total of 116 patients with gastric cancer who underwent gastrectomy and 31 healthy volunteers were prospectively included between 2014 and 2015. Peripheral blood samples were collected before gastrectomy, and CTCs were examined using a centrifugal microfluidic system with a new fluid-assisted separation technique.

**Results:**

After creating a receiver operating characteristic curve to identify the discriminative CTC value needed differentiate patients with gastric cancer from healthy volunteers, sensitivity and specificity were nearly optimized at a CTC threshold of 2 per 7.5 mL of blood. Of the 102 persons with a CTC level ≥2 per 7.5 mL of blood, 99 (97.1%) had gastric cancer, and of the 45 persons with a CTC level <2 per 7.5 mL of blood, 28 (62.2%) were healthy controls. Accordingly, the sensitivity and specificity for the differentiation of patients with gastric cancer from healthy controls were 85.3% and 90.3%, respectively. However, the presence of CTCs was not associated with any clinicopathologic features such as staging, histologic type, or mucin phenotype.

**Conclusion:**

Although we could not prove the clinical feasibility of CTCs for gastric cancer staging, our results suggest a potential role of CTCs as an early diagnostic biomarker of gastric cancer.

## Introduction

Gastric cancer is the fifth-most common malignancy and second-leading cause of cancer-related death worldwide [1]. Although advances in surgery and adjuvant chemotherapy have improved the clinical prognoses of patients with gastric cancer [2], the 5-year survival rate remains <30%, and affected patients are hindered by recurrence and distant metastasis [3].

Circulating tumor cells (CTCs) have been detected in blood from patients with gastric cancer [4–6]. These cells may provide useful prognostic information after surgery or chemotherapy, and may serve as an early cancer diagnostic marker. Previous reports have described various CTC detection methods based on molecular approaches, such as reverse transcriptase-polymerase chain reaction (RT-PCR) analysis, in patients with gastric cancer [7]. However, the ideal CTC detection method should focus directly on tumor cells rather than indirectly linked surrogate markers via a molecular approach [8, 9].

To identify CTCs directly in blood, we recently developed a centrifugal microfluidic system based on a new fluid-assisted separation technique (FAST), in which size-based separation occurs within a centrifugal microfluidic device at a liquid-liquid interface, instead of the conventional liquid-gas interface [10, 11]. In a previous study, we showed that FAST enabled the highly sensitive, selective, rapid, and label-free isolation of CTCs from whole blood without prior sample treatment [10].

Although several studies have shown that CTCs can facilitate gastric cancer diagnosis, prognostic evaluation, and anti-cancer therapy response monitoring [5, 6, 12, 13], the clinicopathologic significance of CTCs remains unclear. Therefore, the present study aimed to evaluate CTCs in patients who underwent gastrectomy for gastric cancer using FAST and to explore the clinical usefulness of these cells in early gastric cancer diagnosis. Furthermore, we evaluated differences in clinicopathologic features according to the level of CTCs.

## Methods

### Clinical study design

Patients with gastric cancer who underwent gastrectomy with lymph node dissection at Pusan National University Hospital (Busan, Korea) were prospectively included. We evaluated the usefulness of CTCs regarding clinicopathologic findings in patients with gastric cancer. A total of 131 consecutive patients with gastric cancer were enrolled at our hospital between August 2014 and December 2015. Fifteen patients were excluded from the analysis, including 12 patients who underwent gastrectomy due to non-curative resection of early gastric cancer by endoscopic submucosal dissection, 2 patients who underwent total gastrectomy for recurrence of gastric cancer after previous subtotal gastrectomy, and 1 patient who had a simultaneous colon cancer. Finally, 116 patients were included in the analysis. Peripheral blood samples were collected before gastrectomy. Written informed consent was obtained from all patients before they underwent blood sampling, and the study protocol was reviewed and approved by the Institutional Review Board of Pusan National University Hospital (H-1412-011-024).

### Surgical procedures

Laparoscopy-assisted or open gastrectomy was performed with lymphadenectomy, resection, and reconstruction [14]. In laparoscopy-assisted gastrectomy, a laparoscope and trocars were inserted through small incisions in the abdominal wall under general anesthesia. Decisions regarding the appropriate range for surgical operation were based on the degree of cancer progression, histopathological diagnosis of the biopsy specimen, tumor location, and risks of lymph node metastasis, morbidity, and mortality. In cases with proximally located tumors, near-total or total gastrectomies were performed using Billroth-I or Billroth-II and Roux-en-Y reconstruction methods. Subtotal gastrectomies were performed for tumors located in the middle third or lower third of the stomach. D1 or D2 lymphadenectomies were performed on a case-by-case basis.

### Histological assessment

The resected specimens were fixed in 10% buffered formalin. Carcinomas and adjacent non-neoplastic mucosal tissues were serially cut into parallel 5-mm sections, embedded in paraffin, sectioned, and stained with hematoxylin and eosin for histological examination. Tumor size, invasion depth, ulceration, degree of differentiation, and lymphovascular invasion were evaluated microscopically according to the Japanese Classification of Gastric Carcinomas [15]. The seventh edition of the American Joint Committee on Cancer classification system was used for tumor-node-metastasis staging [16].

Immunohistochemical expression levels of MUC2 (Ccp58, 1:500; Novocastra Laboratories, Newcastle, UK), MUC5AC (CLH2, 1:500; Novocastra Laboratories), MUC6 (CLH5, 1:500; Novocastra Laboratories), and CD10 (56C6, 1:100; Novocastra Laboratories) in cancer cells were evaluated [17]. Briefly, 5-μm-thick consecutive tumor sections were deparaffinized and hydrated through a graded series of alcohol concentrations. Antigen retrieval was performed in 10 mmol/L citrate buffer (pH 6.0) in a microwave oven for 10 min, after which endogenous peroxidase activity was inhibited by immersing the sections in a 3% H_2_O_2_/methanol solution. The sections were incubated with primary antibodies and thoroughly washed in phosphate-buffered saline (PBS). Next, sections were incubated successively with biotinylated secondary antibody and avidin-biotinylated horseradish peroxidase complex (Vectastain Elite ABC kit; Vector Laboratories30, Burlingame, CA, USA). Antibody binding was visualized using 3,3’-diaminobenzidine tetrachloride as the chromogen, and nuclei were counterstained with Mayer’s hematoxylin. Immunostaining was considered positive if ≥10% of the cancer cells were immunoreactive [17, 18]. Mucin phenotypes were classified into 4 types, gastric, intestinal, gastrointestinal, and null, depending on the expression of gastric mucins (MUC5AC and MUC6) and intestinal mucins (MUC2 and CD10). MUC5AC, MUC6, MUC2, and CD10 are specifically expressed in the gastric foveolar epithelium, pyloric gland cells, goblet cells, and brush border, respectively [19].

### Isolation and enumeration of circulating tumor cells

Three to five-milliliter blood samples were collected in K2-EDTA tubes after discarding the first 2 mL of blood to avoid contamination with skin epithelial cells; each sample was inverted 10 times immediately after collection. To prevent cell damage, blood samples were used within 8 hr. All evaluations were performed by technical assistants without knowledge of the patients’ clinical statuses.

The “FAST disc” centrifugal microfluidic system, which conducts size-selective CTC isolation through membrane pores filled with a stably held liquid throughout the filtration process, was used to detect CTCs [10, 11]. FAST disc isolation of CTCs allows the use of whole blood without any sample treatment steps (e.g., dilution and red blood cell lysis).

Before CTC isolation, the disk surface was passivated with bovine serum albumin; a 1% bovine serum albumin solution was injected into the disc and removed to the waste chamber after 30-min incubation. Next, the disc surface was washed with 1 mL of PBS. After surface passivation, 3 mL of unprepared whole blood was introduced to the disc to isolate CTCs. CTCs were isolated on the membrane by rotating the disc using a programmed spin program. The total process time, including whole blood filtration and filter washing with PBS, was less than 1.0 min.

**Fig 1:**
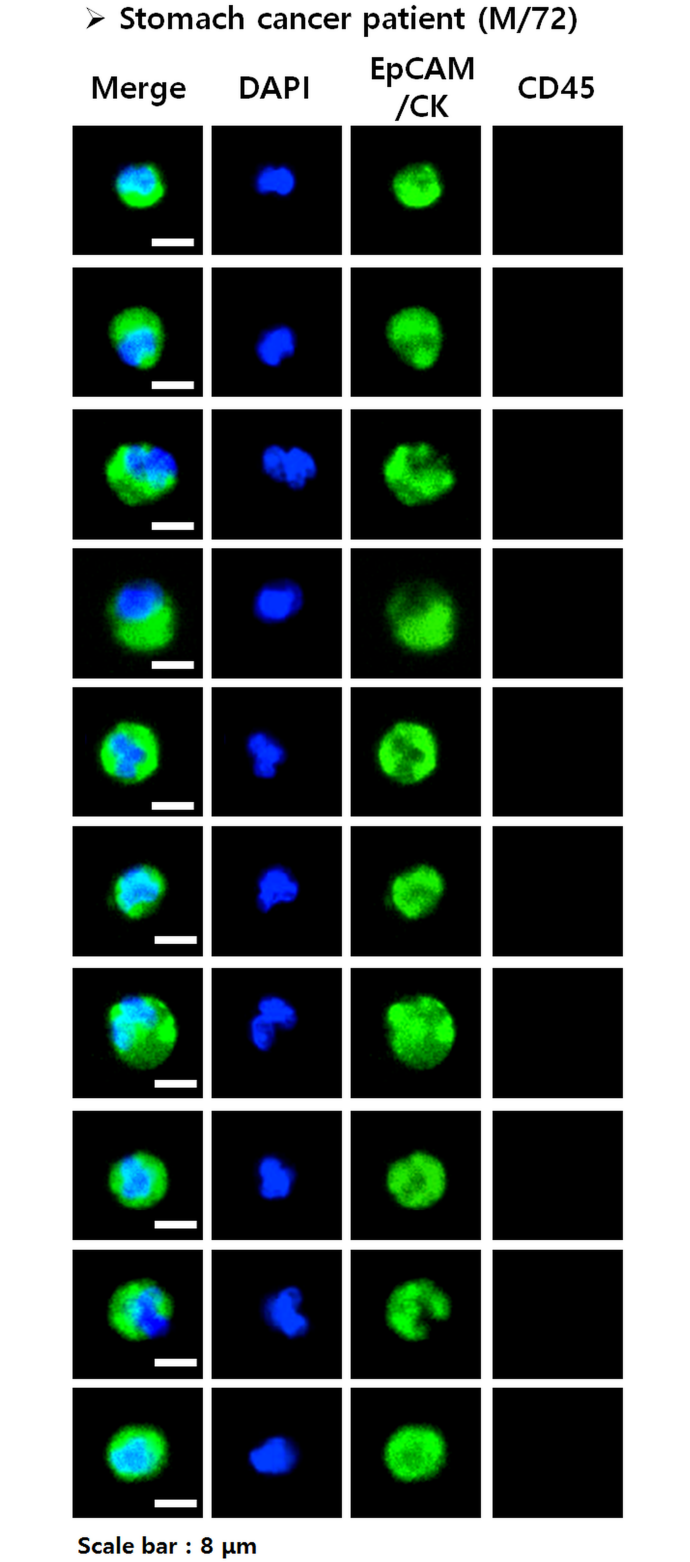
Circulating tumor cells (CTCs). CTCs were defined as captured cells that were CK+ or EpCAM+, CD45-, and DAPI+ and had a diameter >8 μm.

To identify the number of isolated CTCs from sample blood, the disc was immunostained. First, captured cells were fixed with 4% formaldehyde for 15 min at room temperature. Fixed cells were permeabilized with 0.1% Triton X-100 in PBS for 5 min and subsequently washed with PBS. Next, samples were blocked with 20 μg/mL IgG, followed by staining with several antibodies. An anti-CD45 solution (H130; Life Technologies, Carlsbad, CA, USA) was applied for 20 min to stain white blood cells, and samples were then washed with 0.01% Tween 20 in PBS. To stain CTCs, a mixture of FITC-conjugated anti-cytokeratin (CK) (CAM5.2; BD, Franklin Lakes, NJ, USA), Alexa 488-conjugated anti-pan-CK (AE1/AE3; eBioscience, San Diego, CA, USA), and FITC-conjugated anti-epithelial cell adhesion molecule (EpCAM) (9C4; BioLegend, San Diego, CA, USA) was used. This mixture was added to the filter, incubated for 20 min, and followed by washing with 0.01% Tween 20 in PBS. Finally, cell nuclei were stained with 4,6-diamidino-2-phenylindole (DAPI). To visualize CTCs on the filter, fluorescent images were taken using an Eclipse Ti-E fluorescence microscope (Nikon, Tokyo, Japan) at 40× magnification. Captured cells were identified as CTCs if they were CK+ or EpCAM+, CD45-, and DAPI+, and their diameter was > 8 μm (Fig 1). Results are expressed as the number of CTCs per 7.5 mL of whole blood.

For the control group, peripheral blood samples were obtained from 31 healthy volunteers who consented to participate. These volunteers had no current illnesses or previous or family histories of cancer.

### Statistical analysis

A receiver operating characteristic (ROC) curve was created to determine the optimal threshold values for sensitivity and specificity that would best differentiate patients with gastric cancer from healthy controls. The sensitivity, specificity, and positive and negative predictive values for the CTC level that would differentiate patients with gastric cancer from healthy controls were expressed with 95% confidence intervals. Differences in clinicopathologic features and mucin expression were evaluated according to CTC level using the chi-square test or Fisher’s exact test. Statistical calculations were performed using IBM SPSS version 21.0 for Windows (IBM Co., Armonk, NY). Results were considered statistically significant when the *P* value was <0.05.

## Results

### Baseline clinicopathologic characteristics of patients with gastric cancer

The clinicopathologic characteristics of the 116 patients who underwent gastrectomy for gastric cancer are summarized in Table 1. The patients included 74 male and 42 female patients, with a median age of 60 years (range, 33–89 years). Twenty-one tumors were located in the upper third, 43 in the middle third, and 52 in the lower third of the stomach. Ninety-seven patients underwent subtotal gastrectomy (laparoscopic in 50 patients, open in 47 patients), and 19 patients underwent total gastrectomy (laparoscopic in 2 patients, open in 17 patients). The median tumor size was 4 cm (range, 2–13 cm). For pathologic diagnoses, 48 tumors were differentiated-type adenocarcinomas, and 68 tumors were undifferentiated-type adenocarcinomas. Regarding T and N stages, 63, 13, 15 and 24 tumors were T1, T2, T3, and T4, respectively, and 69, 15, 15, and 17 tumors were N0, N1, N2, and N3, respectively. No patients had distant metastases at the time of surgery. Lymphatic invasion, vascular invasion, and perineural invasion were present in 44, 17, and 42 tumors, respectively. Regarding mucin phenotype, 60, 14, 24, and 18 tumors were gastric type, gastrointestinal type, intestinal type, and null type, respectively.

**Table 1:** Baseline clinicopathologic characteristics of 116 patients with gastric cancer.

Median age, years (range)	60 (33–89)
Sex, n (%)	
Male	74 (64)
Female	42 (36)
Location, n (%)	
Upper third	21 (18)
Middle third	43 (37)
Lower third	52 (45)
Histologic type, n (%)	
Differentiated type	48 (41)
Undifferentiated type	68 (59)
Median size, cm (range)	4 (2–13)
T stage, n (%)	
T1	63 (54)
T2	13 (11)
T3	15 (13)
T4	25 (22)
N stage, n (%)	
N0	69 (59)
N1	15 (13)
N2	15 (13)
N3	17 (15)
Lymphatic invasion, n (%)	
Absent	72 (62)
Present	44 (38)
Vascular invasion, n (%)	
Absent	99 (85)
Present	17 (15)
Perineural invasion, n (%)	
Absent	74 (64)
Present	42 (36)
Mucin phenotype, n (%)	
Gastric type	60 (52)
Intestinal type	14 (12)
Gastrointestinal type	24 (21)
Null type	18 (15)

### Thresholds for differentiating patients with gastric cancer from healthy controls

**Fig 2:**
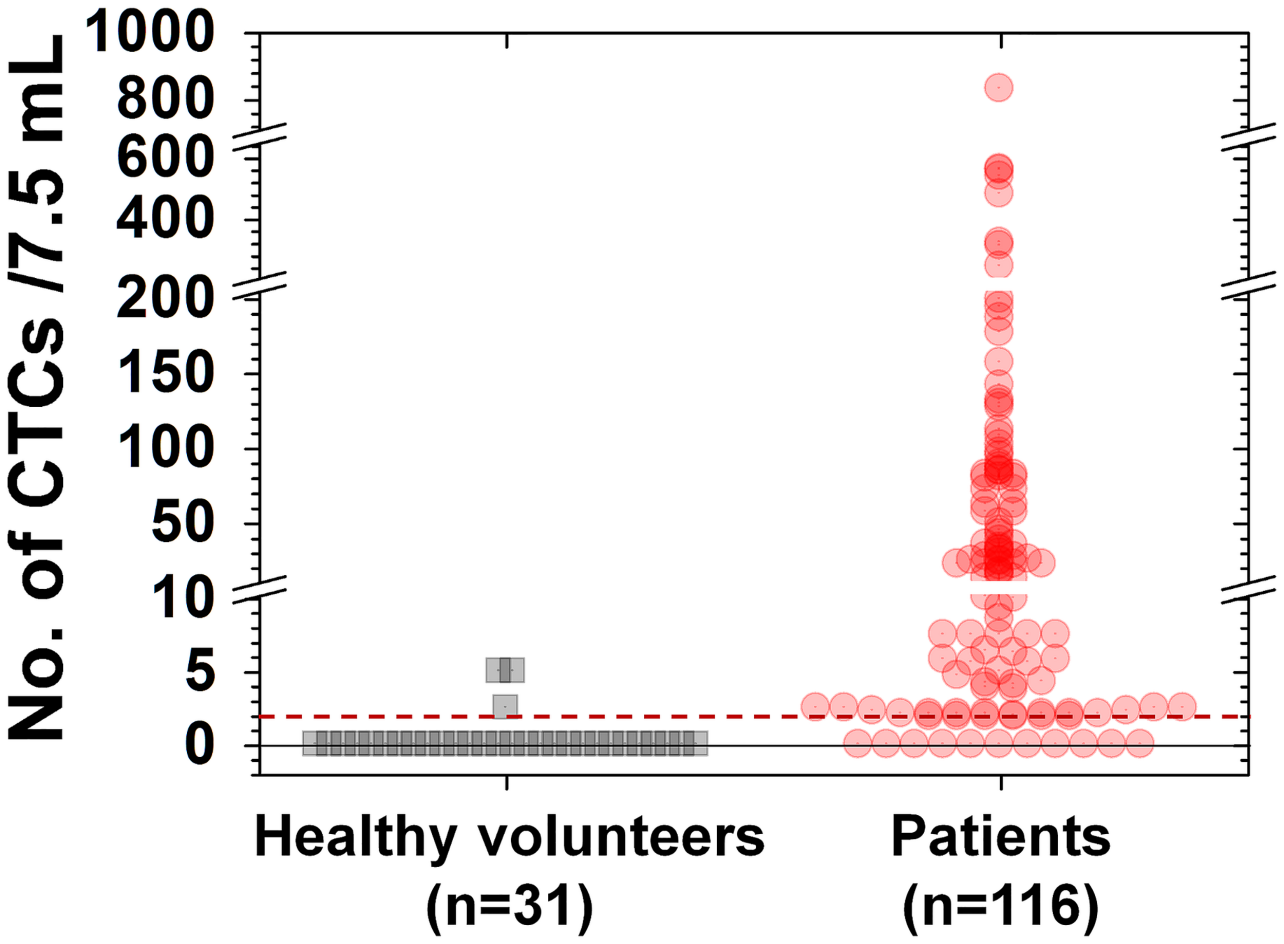
Circulating tumor cells (CTCs) in healthy controls and patients with gastric cancer. CTCs were identified in 3 of 31 healthy controls and 105 of 116 patients with gastric cancer.

Fig 2 shows a scatter plot of CTC values from healthy controls and patients with gastric cancer. CTCs were identified in 3 of 31 healthy controls (CTC counts: 2.5, 5, and 5 per 7.5 mL of blood). In contrast, CTCs were identified in 105 (91%) of 116 patients with gastric cancer. The median CTC count among these patients was 19.5 (range, 0–835) per 7.5 mL of blood.

**Fig 3:**
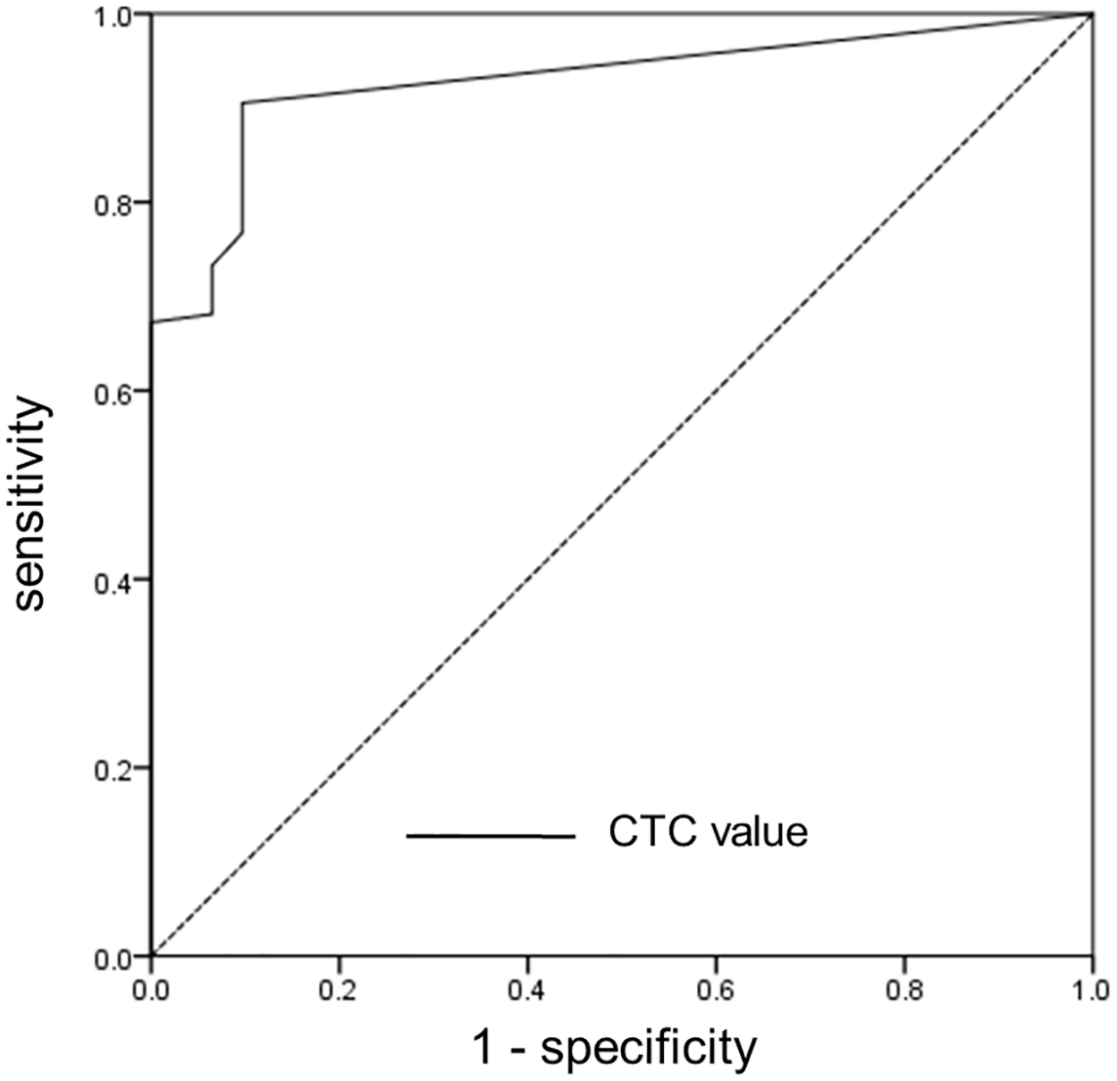
Receiver operating characteristic (ROC) curve. The value of circulating tumor cells (CTCs) showed an area under the ROC curve of 0.928 (95% confidence interval: 0.885–0.971, *P* < 0.001). To identify the optimal CTC threshold value for differentiating patients with gastric cancer from healthy controls, the sensitivity and specificity were optimized using a threshold count of 2 CTCs per 7.5 mL of blood.

A ROC curve was created to identify the optimal CTC threshold value for differentiating patients with gastric cancer from healthy controls (Fig 3). Sensitivity and specificity were optimized using a threshold CTC count of 2 per 7.5 mL of blood. Of the 102 persons with a CTC level ≥2 per 7.5 mL of blood, 99 (97.1%) had gastric cancer. However, of the 45 persons with a CTC level <2 per 7.5 mL of blood, 28 (62.2%) were healthy controls. Therefore, for differentiating patients with gastric cancer from healthy controls, this model had a positive predictive value of 97.1% and negative predictive value of 62.2%, resulting in a sensitivity of 85.3% and specificity of 90.3% (Table 2).

**Table 2:** Sensitivity, specificity, and positive and negative predictive values of circulating tumor cells for the differentiation of patients with gastric cancer from healthy controls.

	Sensitivity (%, 95% CI)	Specificity (%, 95% CI)	Positive predictive value (%, 95% CI)	Negative predictive value (%, 95% CI)
CTC ≥2 per 7.5 mL of blood	85.3 (81.4–87.2)	90.3 (75.6–97.4)	97.1 (92.6–99.2)	62.2 (52.1–67.1)

CTC, circulating tumor cell; CI, confidence interval.

### Association between circulating tumor cells and clinicopathologic findings in gastric cancer

Of the 116 patients with gastric cancer, 99 (85%) had ≥2 CTCs per 7.5 mL of blood. CTCs were not found to associate with histologic type or tumor size (*P* = 0.985 and *P* = 0.429, respectively; Table 3). Although CTCs did not associate with T or N stage (*P* = 0.581 and *P* = 0.510, respectively), CTCs were detected in >80% of patients with T1-stage and N0-stage disease. CTCs were also not found to associate with lymphatic or vascular invasion (*P* = 0.713 and *P* = 0.765, respectively).

**Table 3:** Clinicopathologic features of patients with gastric cancer according to the level of circulating tumor cells.

	CTC <2 (n = 17)	CTC ≥2 (n = 99)	*P*-value
Sex, n (%)			0.120
Men	8 (11)	66 (89)	
Women	9 (21)	33 (79)	
Age, n (%)			0.216
≤60 years	11 (19)	48 (81)	
>60 years	6 (11)	51 (89)	
Location, n (%)			0.008
Upper third	4 (19)	17 (81)	
Middle third	1 (2)	42 (98)	
Lower third	12 (23)	40 (77)	
Histologic type, n (%)			0.985
Differentiated type	7 (15)	41 (85)	
Undifferentiated type	10 (15)	58 (85)	
Tumor size (cm, mean ± SD)	5.2 ± 3.2	4.6 ± 2.7	0.429
T stage, n (%)			0.581
T1	7 (11)	56 (89)	
T2	2 (15)	11 (85)	
T3	3 (20)	12 (80)	
T4	5 (20)	20 (80)	
N stage, n (%)			0.510
N0	8 (12)	61 (88)	
N1	2 (13)	13 (87)	
N2	3 (20)	12 (80)	
N3	4 (24)	13 (76)	
Lymphatic invasion, n (%)			0.713
Absent	14 (14)	85 (86)	
Present	3 (18)	14 (82)	
Vascular invasion, n (%)			0.765
Absent	10 (14)	62 (86)	
Present	7 (16)	37 (84)	
Perineural invasion, n (%)			0.314
Absent	9 (12)	65 (88)	
Present	8 (19)	34 (81)	

CTC, circulating tumor cell; SD, standard deviation.

### Association between circulating tumor cells and mucin phenotypes in gastric cancer

MUC2, MUC5AC, MUC6, and CD10 expression levels were increased in 32 (28%), 80 (69%), 28 (24%), and 12 tumors (10%), respectively. CTCs did not associate with mucin expression (Table 4). CTCs were detected in 53 (88%) of 60 gastric type, 21 (87%) of 24 intestinal type, 11 (79%) of 14 gastrointestinal cancers, and 14 (78%) of 18 null type cancers. There were no differences in CTCs according to mucin phenotype (*P* = 0.606).

**Table 4:** Mucin expression in gastric cancer according to the level of circulating tumor cells.

	CTC <2 (n = 17)	CTC ≥2 (n = 99)	*P*-value
MUC2 expression, n (%)			1.000
Negative	12 (14)	72 (86)	
Positive	5 (16)	27 (84)	
MUC5AC expression, n (%)			0.397
Negative	7 (19)	29 (81)	
Positive	10 (12)	70 (88)	
MUC6 expression, n (%)			1.000
Negative	13 (15)	75 (85)	
Positive	4 (14)	24 (86)	
CD10 expression, n (%)			1.000
Negative	16 (15)	88 (85)	
Positive	1 (8)	11 (92)	
Mucin phenotype, n (%)			0.606
Gastric type	7 (12)	53 (88)	
Intestinal type	3 (13)	21 (87)	
Gastrointestinal type	3 (21)	11 (79)	
Null type	4 (22)	14 (78)	

CTC, circulating tumor cell.

## Discussion

In the present study, CTCs were identified in 105 (91%) of 116 patients with gastric cancer, and a threshold of ≥2 CTCs per 7.5 mL of blood was useful for differentiating patients with gastric cancer from healthy controls. However, no associations were observed between CTCs and clinicopathologic features such as histologic type, T stage, N stage, and mucin phenotype. To our knowledge, this is the first report to demonstrate FAST-based CTC detection as an early diagnostic biomarker for gastric cancer.

Numerous reports have described the isolation and characterization of CTCs from patients with gastric cancer [20]. Because CTCs have been observed at very low concentrations (10^−7^–10^−8^) relative to normal peripheral blood cells in samples from patients with cancer [21], CTC detection in blood requires highly sensitive, specific, and reproducible methods. Immunocytochemistry, RT-PCR procedures, flow cytometry, and CellSearch system have all been used to detect these rare CTCs [20]. Of these, the CellSearch system is the first and only FDA-approved test applicable for CTC detection in patients with metastatic breast, prostate, or colorectal cancer. This system is based on the enumeration of epithelial cells, which are separated from the blood using EpCAM antibody-coated magnetic beads and identified with fluorescently labeled antibodies against cytokeratin and a fluorescent nuclear stain. However, the CellSearch system has several shortcomings, including an inability to capture and analyze cells lacking tumor marker expression, poorly differentiated cells, or circulating tumor stem cells [22]. Therefore, other CTC acquisition methods have been pursed, including isolation by size, in which tumor cells are isolated and individually filtered from blood cells based on the differential size of epithelial cells, have been pursued [23]. Accordingly, many researchers have developed lithography-based microfilters with controlled pore sizes and shapes to increase porosity and thus enhance isolation [24–26]. However, these methods require blood samples to be diluted and/or fixed to achieve higher recovery and avoid frequent clogging. More importantly, these methods are not suitable for mass production [27, 28]. Commercial size-based CTC isolation kits utilize less expensive track-etched polycarbonate membranes instead of lithography-based microfilters. However, the non-uniform and randomly distributed pores and low porosity of these membranes can cause inconsistent and lower-than-desired recoveries and purity [28, 29]. Here, we used a centrifugal microfluidic device operated in FAST mode to overcome the limitations of track-etched based filtration using a hydrodynamic approach [10]. FAST provides uniform, clog-free, and efficient filtration under a lower pressure drop when compared to conventional separation, leading to highly sensitive (>95.9% recovery), selective (~2.5 log depletion of white blood cells), rapid (>3 mL/min), and label-free isolation of CTCs from whole blood without prior sample treatment [10, 11].

In the present study, CTCs were identified in 3 (9.6%) of 31 healthy controls, consistent with the results of a previous study, in which CTCs were detected in 8 (5.5%) of 145 healthy controls [30]. Of the 116 patients with gastric cancer, CTCs were identified in 105 (91%) patients, higher than the positive rates (10.8–69%) reported previously by using the CellSearch system [5, 6, 31]. We used a ROC curve to identify the optimal threshold level of CTCs for differentiating patients with gastric cancer from healthy controls, and defined this threshold as ≥2 CTCs per 7.5 mL of blood, similar to previous reports [30, 31]. Using this threshold, 99 (85%) of 116 patients with gastric cancer were found to have ≥2 CTCs per 7.5 mL of blood, and this rate was similar irrespective of T stage and N stage. Overall, the sensitivity and specificity values for the threshold of 2 CTCs per 7.5 mL as a differentiator of patients with gastric cancer from healthy controls were 85.3% and 90.3%, respectively. These results suggest the potential use of CTCs as an early diagnostic biomarker of gastric cancer.

A recent meta-analysis of CTCs in patients with gastric cancer suggested correlations of CTCs with tumor staging, histologic type, and lymphovascular invasion [20]. However, most studies included in that meta-analysis detected CTCs using RT-PCR or immunohistochemistry; only 2 studies used the CellSearch system. In those latter studies, however, CTCs were found to correlate with tumor staging and lymphovascular invasion [5, 31]. In contrast, the present study found no associations of the CTC level with clinicopathologic features. The difference between our results and those of previous studies might be attributable to heterogeneity in the baseline clinicopathologic characteristics and the use of different CTC detection methods.

As shown in this report, CTCs can be detected in cancer patients without clinically detectable metastasis [32]. However, not all CTCs have the potential to develop into metastases; only a small subset with stem cell-like or epithelial-mesenchymal transition properties can survive and migrate to distant sites to establish secondary tumors [33]. Therefore, the subclassification of detected CTCs using stem cell or epithelial-mesenchymal transition markers would provide more useful information for tumor stage evaluation and prediction of recurrence and metastasis. Furthermore, CTC monitoring could be useful for predicting tumor progression, prognosis, and the effects of chemotherapy in patients with gastric cancer [5, 6]. Therefore, FAST-based CTC measurement is advantageous because it can be performed easily and noninvasively at any time.

Nonetheless, this study has several limitations. First, because we focused on the presence of CTCs at the time of surgery, we did not examine long-term follow-up results, such as recurrence and survival, based on the level of CTCs. Secondly, we attempted to associate CTCs with clinicopathologic findings based on the final pathologic results and therefore did not include patients with distant metastases. In the future, we plan to analyze the roles of CTCs in predicting the long-term (at least 2 years) outcomes and responses to chemotherapy in patients with distant metastases of gastric cancer. Thirdly, because a loss of epithelial markers can occur during progression to malignancy [34], the use of an EpCAM antibody-based CTC detection method creates a potential limitation to the sensitivity of the current generation of CTC detection platforms [35].

## Conclusions

Although the clinical feasibility of CTC assessment for gastric cancer staging was not proven, our study results suggest a potential role of FAST-based CTC detection as an early diagnostic biomarker of gastric cancer. These results should be further supported by additional large, prospective multi-center studies. In addition, the role of CTCs as a biomarker predictive of patients’ prognoses and responses to chemotherapy should be investigated during a long-term follow-up.

## Supporting information

S1 File Approved document by the Institutional Review Board. (PDF)
