# Supplementary material for: Circulating tumor cells detected by lab-on-a-disc: Role in early diagnosis of gastric cancer
Source: PLoS One. 2017 Jun 29;12(6):e0180251. doi: 10.1371/journal.pone.0180251 (PMC5491173; doi:10.1371/journal.pone.0180251)
Supplement: S1 File — (PDF) [file pone.0180251.s001.pdf]

수신자 : 부산대학교병원

제목: 의학연구윤리심의위원회(IRB) 심의 결과 보고

1. 본 기관 의학연구윤리심의위원회(IRB)에서 아래와 같이 심의하여 결과를 통보하여드립니다.

가. IRB NO. :   H-1412-011-024

나. 연구 책임자 :   김광하 교수

다. 연구 과제명 : 위암 및 대장암 환자에서 혈액 순환 암세포의 유용성 평가

라. 심의 대상 :   연구계획심의 의뢰서

붙임: 의학연구윤리심의위원회(IRB) 심의결과통보서. 끝

부산대학교병원장

시행 연구윤리심의위원회 -   1412-006-001(2014.12.30) 접수 (   )

우 49241 부산광역시 서구 구덕로 179 부산대학교병원 /

전화 051-240-7000 / 전송 / / 공개

통지서

수신	의뢰기관	부산대학교병원		
	연구책임자	(소속) 소화기내과 (직위) 교수 (성명) 김광하		
IRB 과제번호	H-H-1412-011-024		심사내용	
연구과제명	위암 및 대장암 환자에서 혈액 순환 암세포의 유용성 평가			
연구대상	□ 의약품 □ 의료기기 □ 식품 □ 화장품 □ 시술법 ■ 기타(진단법)			
	일반명<br>(분류코드)	NA	상품명	NA
연구예정기간	2014 년 12 월 30 일 ~ 2019 년 12 월 31 일			
최초승인일자	2014년 12월 30일 (정기보고주기 : 12개월)			
승인기간	2014년 12월 30일 ~ 2015년 12월 29일			
심의종류	정규심의		심의대상	연구계획심의 의뢰서
접수일자	2014년 12월 15일		심의일자	2014년 12월 30일
심의자료	1. 임상시험 심의 의뢰서<br>2. 프로토콜요약자료<br>3. 임상시험계획서(Version1.0)<br>4. 증례기록서(Version1.0)<br>5. 대상자 동의서 및 설명문(Version1.0)<br>6. 연구자 이력서 및 교육이수증<br>7. 임상시험자서약서<br>8. 경제적 이해갈등관계내역<br>9. 연구비내역서<br>10.대상자 보상규약			
심의결과	승인			
심의의견				
중간보고여부	■ 예 (보고시기 : 2015-12-29) □ 아니오			

※ 본 심사위원회는 ICH-GCP 및 KGCP를 준수하며, 생명윤리및안전에관한법률 등 관련법규를 준수합니다.

※ 이해갈등 관련 IRB 위원이 있는 경우 토론 및 결정과정에 참여하지 못하도록 위원장이 조치하였습니다.

※ 만약 본 위원회의 심의결과에 불복할 경우, 심의결과 통보 후 그 사유를 기록하여 이의를 신청할 수 있습니다.

※ 본 심사위원회에서 통지한 대로 연구기간 1년마다 지속심의의뢰서를,중간보고 기간에 중간보고서를 연구 종료시에는 종료보고서를 제출해주시고 종료보고서 제출 후 1년 안에 결과보고서를 제출해 주시기 바랍니다.

※ 연구 중에 중대한 이상반응 발생 시 연구책임자는 본 위원회에 즉시 보고해야 합니다.

※ 본 임상연구 결과를 임상시험실시기관의 사전 서면동의 없이는 어떤 경우라도 학술목적 이외에 실시기관명을 사용할 수 없습니다.

※ 본 통지서는 KGCP 제 13조 1항에 따른 심사 통보서로 사용할 수 있습니다.

※ 심사위원회의 결정사항에 대해 이의가 있는 경우 이의신청 절차에 따라 2회까지 이의신청이 가능합니다.

부 산 대 학 교 병 원
임 상 시 험 심 사 위 원 회
